# The pandemic (H1N1) 2009 influenza virus is resistant to mannose-binding lectin

**DOI:** 10.1186/1743-422X-8-50

**Published:** 2011-02-04

**Authors:** Hirotoshi Tokunaga, Hiroshi Ushirogawa, Masanobu Ohuchi

**Affiliations:** 1 Department of Microbiology, Kawasaki Medical School, 577 Matsushima, Kurashiki, Okayama 701-0192, Japan; 2 Division of Hematology, Department of Medicine, Kawasaki Medical School, 577 Matsushima, Kurashiki, Okayama 701-0192, Japan

## Abstract

**Background:**

Mannose-binding lectin (MBL) is an important component of innate immunity because it promotes bacterial clearance and neutralization of human influenza A viruses. Since a majority of humans have no neutralizing antibody against the pandemic (H1N1) 2009 influenza (pandemic 2009) virus, innate immunity may be crucial and MBL susceptibility may therefore influence viral pathogenesis.

**Results:**

We examined MBL susceptibility of influenza A viruses and observed that the pandemic 2009 virus was resistant to MBL, whereas all seasonal influenza A viruses tested were susceptible. The mortality of mice infected with a seasonal H1N1 influenza virus was evidently enhanced on transient blockage of MBL activity by simultaneous inoculation of mannan, whereas mannan inoculation had no effect on mice infected with a pandemic 2009 virus. This indicates that MBL protects mice against infection with the seasonal virus but not against that with the pandemic 2009 virus.

**Conclusions:**

These results indicate that the pandemic 2009 virus is not susceptible to MBL, an important component of innate immunity.

## Background

A novel influenza virus of swine origin, which emerged in North America in 2009, rapidly spread worldwide and caused the influenza pandemic 2009. This virus was classified as type A and subtype H1N1 according to the antigenicity of hemagglutinin (HA) and neuraminidase proteins [1]. Currently, the pandemic 2009 caused due to influenza H1N1 virus has ceased. However, in case of the 1918 Spanish flu pandemic, low mortality was observed at the first wave, followed by a second wave that caused a severe pandemic with high mortality [2,3]. Whether a second wave of the pandemic (H1N1) 2009 will emerge is difficult to predict. Pathogenesis of the pandemic (H1N1) 2009 influenza (pandemic 2009) virus has not yet been completely elucidated. Pathogenesis of a virus is not only determined by its ability to infect and grow in its host but also by its interaction with host defense mechanisms. Prior to an acquired immune response, especially in case of primary infection with a foreign pathogen, innate immunity is crucial for host defense. Consequently, the susceptibility of a pathogen to an innate immune response inevitably determines its pathogenesis. Human seasonal influenza A viruses are susceptible to mannan-binding protein, also known as mannose-binding lectin (MBL) [4-9], which is an acute protein produced by the liver [10,11]. Usually, the blood MBL level may not be sufficiently high to directly inhibit initial infection with the influenza virus. However, when MBL production is upregulated in response to inflammation, MBL may restrict the development of viral infection in the host. Thus, it is expected to function as a "brake" towards inhibiting the viral propagation. Therefore, MBL susceptibility of the pandemic 2009 virus must be determined in order to discuss the pathogenesis of this virus and the potential toward causing a severe second wave of pandemic.

## Results

### MBL susceptibility of the pandemic 2009 virus

**Figure 1:**
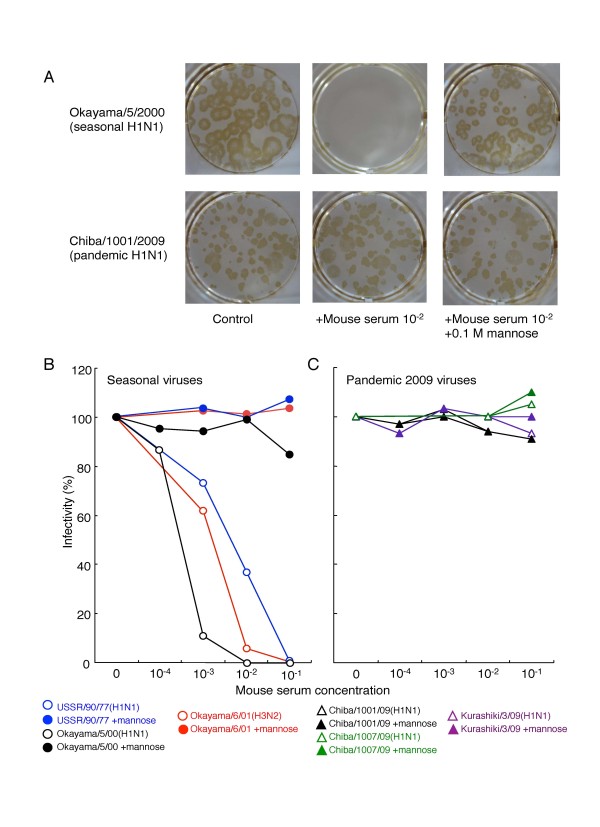
**MBL Susceptibility of seasonal and pandemic 2009 viruses**. (A) Plaques were visualized by immunostaining. To eliminate the effects of MBL, the mouse serum was diluted with DMEM containing 0.1 M mannose. (B and C) Inhibitory effect of mouse serum on seasonal and pandemic 2009 influenza viruses. The infectivity of each specimen was expressed as a percentage of the plaque number of the control (without mouse serum). Each value is the mean derived from two independent experiments.

We compared MBL susceptibility of the seasonal and pandemic viruses using normal mouse sera, because of the following reasons. First, human-derived products such as human MBL are not commercially available. Second, most human serum contains a high titer of neutralizing antibodies against the seasonal influenza viruses, whereas the MBL content is usually low. Third, mouse serum contains a high titer of MBL and the sugar recognition specificity of murine MBL closely resembles that of human MBL [5,7,12,13], and the minimum concentration of murine MBL required to generate anti-influenza neutralizing activity almost closely resembles that of human MBL [4,6,8,9]. Sera from naive C57BL/10 mice were serially diluted 10-fold with Dulbecco's modified Eagle's medium (DMEM), and this mixture was then mixed with a virus suspension containing approximately 1 × 10^2 ^plaque-forming units (PFU) of the seasonal or the pandemic 2009 viruses, and then incubated for 30 min at room temperature. The mixtures were then inoculated into Madin-Darby canine kidney (MDCK) cell cultures and assayed for viral infectivity. Representative pictures of the plaque assay are shown in Figure 1A. Plaques (represent infectivity) of the seasonal A/Okayama/5/2000 (H1N1) virus were evidently reduced when the virus suspension was pretreated with 1:100 diluted mice sera, whereas plaques of the pandemic 2009 A/Chiba/1001/2009 virus were not affected by the same pretreatment. The inhibitory effect of the mice sera on the seasonal Okayama virus was completely eliminated by the addition of mannose to the pretreatment mixture, which clearly demonstrates that the effect was mediated by MBL. The inhibitory effects of the mice sera on the seasonal and pandemic 2009 viruses are shown in Figure 1B and Figure 1C, respectively. All three seasonal influenza A viruses were evidently neutralized with murine MBL, whereas the pandemic 2009 viruses tested did not show any MBL susceptibility. Similar results were obtained in experiments with BALB/c mice sera and other seasonal and pandemic 2009 strains (data not shown). These results demonstrate that the pandemic 2009 virus lacks MBL susceptibility.

### Glycosylation site of the HA protein responsible for MBL susceptibility

**Figure 2:**
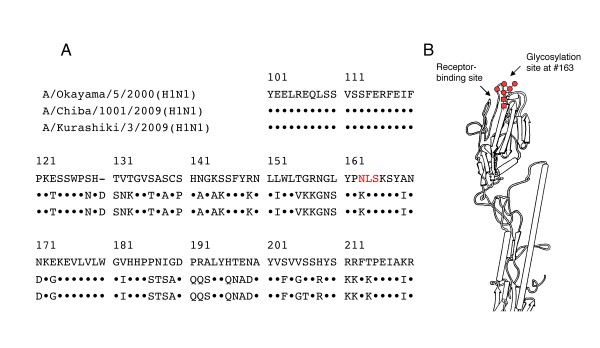
**Glycosylation motif around amino acid 165 of the HA protein of seasonal H1N1 and pandemic 2009 viruses**. (A) Amino acid sequence. Okayama/5/2000, seasonal virus; Chiba/1001/2009 and Kurashiki/3/2009, pandemic 2009 viruses. ● indicates the same amino acid as that in Okayama/5/2000. Red letters indicate *N*-glycosylation motif. (B) The deduced location of the glycosylation site in the 3D model of the HA protein [14].

The HA protein of the human influenza H3N2 virus displays a mannose-rich glycan at the *N*-glycosylation site of amino acid 165 (asparagine) [14]. MBL binds to this glycan and because of its proximity to the receptor-binding site; MBL inhibits viral access to the receptors [4,7]. This glycosylation motif is highly conserved in all human H3N2 viruses. In order to determine the presence of such a motif in the HA protein of the seasonal and pandemic H1N1 viruses tested in this study, we directly sequenced HA genes of the seasonal Okayama/5/2000 as well as pandemic Chiba/1001/2009, and Kurashiki/3/2009 viruses. As shown in Figure 2A, the seasonal Okayama virus has a glycosylation motif at amino acid 163 (H3 alignment numbering), whereas the pandemic Chiba/1001 and Kurashiki/3 viruses have no glycosylation motif in this region. The same pattern has been observed in almost all other seasonal and pandemic H1N1 viruses [15]. This amino acid 163 site of the H1 HA protein has shown to be glycosylated [16]. The deduced location of the glycan is shown in Figure 2B. The infectivity of seasonal influenza A viruses can be neutralized by the binding of MBL to the glycan in this region.

### Contribution of MBL to host defense in mice infected with seasonal or pandemic 2009 viruses

**Figure 3:**
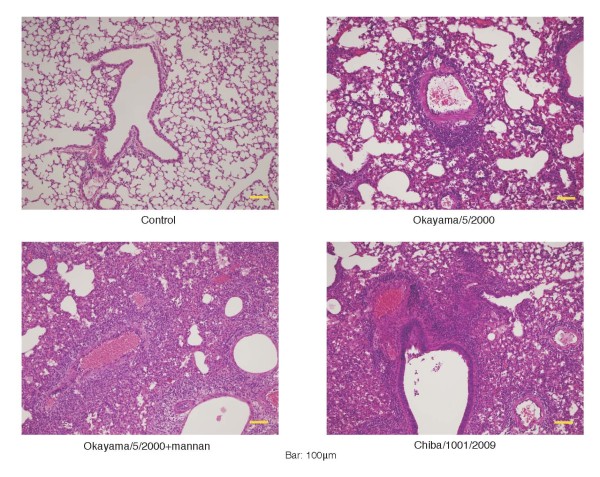
**Histopathological observation of the lungs of virus-inoculated mice**. On day 8 after infection, the lungs were removed from the mice under anesthesia and subjected to histopathological examination. Lung section of the mouse co-inoculated with mannan is also shown.

**Figure 4:**
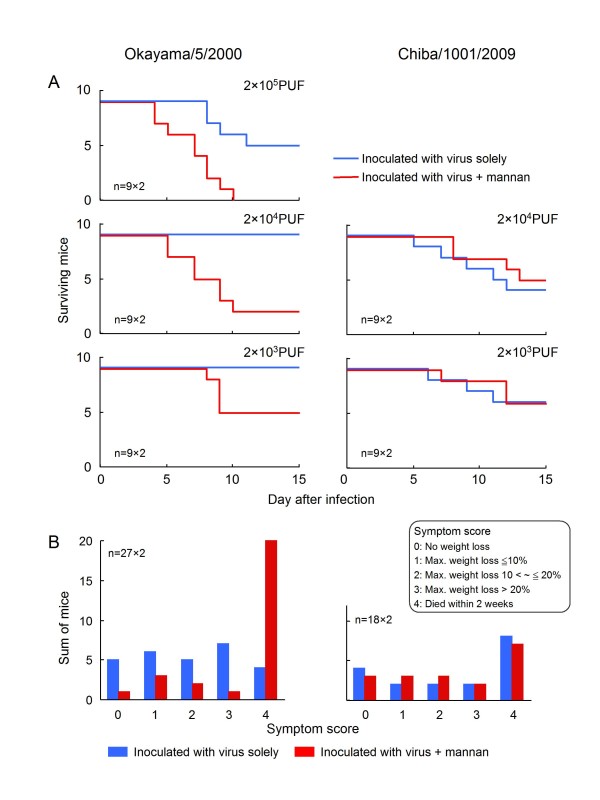
**Effect of mannan inoculation on influenza virus infection in mice**. (A) Mortality of mice infected with seasonal or pandemic 2009 viruses. Each mouse was intranasally inoculated with 20 μl of virus suspension (containing 0% or 1% mannan) under anesthesia. (B) The severity of infection in each mouse was scored according to the maximum loss of body weight (% of weight on day 0 after infection), and mice with the same score were summed together.

We confirmed the MBL susceptibility of seasonal and pandemic viruses in a mouse infection model. Although, the MBL level in mouse serum is always high [4], intranasal inoculation of mannan in the mouse respiratory tract transiently blocks the MBL activity [8]. The blocking of MBL activity considerably affects the progress of viral infection in cases where the virus is susceptible to MBL. If the virus is resistant to MBL, the progress of infection is not affected by mannan inoculation. Mice were intranasally inoculated with 20 μl of virus suspension with or without 1% mannan. After infection, the body weight of the mice was monitored daily until day 28. Histopathological examination of the lungs of the infected mice with weight loss revealed pneumonia with cellular infiltration (Figure 3). Mortality of the infected mice is shown in Figure 4A. Mortality in mice infected with the seasonal Okayama/5/2000(H1N1) virus evidently increased on simultaneous inoculation of 1% mannan, whereas such an effect was not observed in those infected with the pandemic 2009 Chiba virus. The severity of infection in each mice were scored according to the maximum loss of body weight (%) [no loss was scored as 0 (no symptom); ≤10% was scored as 1 (mild); >10% to ≤20% was scored as 2 (moderate); >20% was scored as 3 (severe); and death was scored as 4 (fatal)]. As shown in Figure 4B, when MBL activity was transiently blocked by simultaneous inoculation of mannan, the symptoms became more severe in the mice infected with the seasonal virus (*p *< 0.001, Welch's two-tailed t-test), whereas no change was observed in those infected with the pandemic 2009 virus. These findings confirm that MBL actually contributes to host defense in mice infected with MBL susceptible viruses, and that the pandemic 2009 virus is resistant to MBL.

## Discussion

Human influenza A viruses excluding mouse-adapted viruses have a glycosylation site at amino acid 165 of the HA protein [14]. Glycan at amino acid 165 of the H3 HA protein is rich in mannose, and is thus responsible for MBL susceptibility of human H3N2 influenza viruses [4,7]. The 1918 Spanish flu virus has no glycosylation site around amino acid 165 of the HA protein [17], and therefore, it might be resistant to MBL. Through reverse genetics, Kobasa et al. substituted the HA protein of a human-origin influenza virus with that of the Spanish flu virus and found that the resulting virus caused severe pneumonia with extensive cellular infiltration in mice [18]. They proposed that the high pathogenicity of this virus was due to an abnormality in the innate immune response of the mice. Accordingly, we suggest that MBL, which is an important component of innate immunity, might fail to neutralize this virus in the mouse lung.

This raises the question as to why the pandemic 2009 virus is not as virulent in humans as the Spanish flu virus. In our experiments with human-origin cell cultures (unpublished data), the growth rate of the pandemic 2009 virus was distinctly lower than that of seasonal H3N2 and H1N1 viruses, suggesting that the pandemic virus may not yet have adapted sufficiently to the human cells. Thus, the pandemic 2009 virus can potentially cause a severe second wave of pandemic, once it acquires such a high growth rate in humans.

Although the advantage of the presence of a mannose-rich glycan at the tip of the HA protein of the virus remains unclear, it may attenuate viral pathogenesis, facilitating patient mobility and thereby contributing to wide dissemination of the virus, even after a large population of humans has obtained immunity against it. By 1933, a descendant of the Spanish flu virus obtained a glycosylation site at amino acid 165 of the HA protein to which a mannose-rich glycan could attach [15]. Since no virus was isolated from humans before 1933, the exact year of this development remains unknown.

In the database of influenza viruses, four pandemic 2009 isolates with the glycosylation motif at amino acid 165 in the HA protein have been reported [19], which were first isolated in Novouralsk, Russia (November 2009) and later in Orenburg, Russia (March 2010). Both of these regions are located in Central Asia, and the nucleotide sequence homology of *HA *is >99.6% among the isolates [20], suggesting that they belong to the same lineage. This lineage may be either extinct or present in an extremely lower number of human populations. Such MBL susceptible viruses may have no competitive advantage in the present situation, wherein a majority of people have no immunity against the virus.

The contribution of MBL to host defense in influenza infection has not yet been completely elucidated. Regarding the innate host defense mechanism in respiratory organ, surfactant protein D (SP-D) and A (SP-A) should be considered as the most potent factors in the defense mechanism [21-23]. Since SP-D is known to show sugar specificity similar to MBL [21], it would be interesting to examine the susceptibility of the pandemic 2009 virus to SP-D and also to SP-A. These factors will now be of utmost importance towards advancing our understanding of influenza pathogenesis. Future studies including extensive clinical studies are urgently required in this area of research.

## Conclusions

The pandemic 2009 influenza virus is not susceptible to MBL, and therefore, the MBL resistance property of this virus may be one of its potential pathogenic factors.

## Methods

### Viruses and cells

Pandemic influenza A/Chiba/1001 and 1007/2009 (H1N1) viruses were provided by Dr. Ogawa T from the Chiba Prefectural Institute of Public Health. The A/Kurashiki/3/2009 (H1N1) virus was isolated in our laboratory. For seasonal influenza viruses, we used A/USSR/90/1977 (H1N1) (provided by Dr. Nobusawa R from the Nagoya City University), A/Okayama/5/2000 (H1N1), and A/Okayama/6/2001 (H3N2) (both isolated in our laboratory). Each virus stock was purified twice by limiting dilutions through MDCK cells grown in a 12-well plate and then propagated once in MDCK cells in a bottle. The passage number of Chiba, Kurashiki, and Okayama strains used in the present study was between 5 and 7. The USSR/77 virus was propagated once in an embryonated chicken egg.

MDCK cells were grown in Eagle's minimum essential medium supplemented with 10% fetal calf serum (FCS). For viral infection, MDCK cells were washed twice with FCS-free medium and then supplemented with fresh DMEM. All viral experiments were performed in a level 2 biosafety laboratory.

### Assay of viral infectivity

MDCK cells propagated in a 12-well culture plate were inoculated with virus suspensions and incubated for 1 h at 37°C, and then the culture medium was replaced with DMEM containing 0.6% agarose and 1 μg/ml acetylated trypsin (Sigma, USA). In agarose-containing medium, trypsin-activated progeny viruses infected only neighboring cells and thus formed a plaque. Accordingly, one PFU was considered to correspond to one infectious virus. Two days after virus inoculation, the plaques were stained with anti-influenza A virus guinea pig antiserum (prepared in our laboratory), and peroxidase-conjugated goat anti-guinea pig IgG (Jackson Immuno Research, USA).

### Neutralization of viral infectivity with murine MBL

Sera were prepared from 3.5- to 7.5-week-old naive C57BL/10slc or BALB/c mice (SLC Laboratory, Japan). In order to measure the virus neutralizing activity, serum was serially diluted 10-fold with DMEM, and then mixed with the virus suspension. To block MBL activity, the serum was diluted with DMEM containing 0.1 M mannose (Sigma, USA). After incubating the mixtures for 30 min at room temperature, they were inoculated into MDCK cell cultures to determine the remaining infectivity.

### Sequence analysis of the region around amino acid 165 in the HA protein

Virus suspension (1.5 ml) was centrifuged at 10,000 × *g *for 90 min at 4°C, and RNA was extracted from the precipitate using Isogen (Wako Chemicals, Japan). *HA *cDNA was synthesized from RNA by RT-PCR using specific primers. The PCR product was subjected to agarose gel electrophoresis, and the specific band was excised from the gel and purified using a QIAquick Gel Extraction Kit (Qiagen, Germany). Sequencing was performed using the same primers as those used in RT-PCR. The primers used for the seasonal Okayama/5/2000 strain were huH1-257 (forward, 5'-TAC TGA TTT CCA AGG AGT C-3') and huH1-885 (reverse, 5'-CTT CGC ATC ACA TTT ATC CAT-3'), and those used for the pandemic Chiba/1001/2009 and Kurashiki/3/2009 strains were swH-257 (forward, 5'-CAC TCT CCA CAG CAA GCT C-3') and swH-885 (reverse, 5'-AGT TGT ATT GCA ATC GTG GAC-3'), respectively. The nucleotide sequences were deposited in the DNA Data Bank of Japan (accession number AB568299-301).

### Experimental infection of mice with seasonal and pandemic viruses

Female 3.5-week-old C57BL/10slc or BALB/c mice were intranasally inoculated with a 20 μl suspension of the seasonal Okayama/5/2000(H1N1) or pandemic 2009 Chiba/1001/09(H1N1) viruses under anesthesia with sevoflurane. The body weight of each mouse was monitored daily. For histopathological examination, lungs were removed from several mice on day 8 after infection. At the end of the experiment, blood samples were collected from the surviving mice under anesthesia and the anti-influenza antibody titer in the serum was assayed to confirm infection; this analysis was performed even for asymptomatic mice.

To temporarily block MBL activity in mice, soluble mannan (Sigma, USA) was added to the virus suspension at a final concentration of 1% (w/v). Severity scores of the infected mice administrated with and without mannan were statistically compared using Welch's two-tailed t-test. This experiment was approved by the Animal Experiment Committee of Kawasaki Medical School (Approval No. 09-066) and performed in a P2A level laboratory.

## Competing interests

The authors declare that they have no competing interests.

## Authors' contributions

HT performed the *in vitro *assays for MBL neutralizing activity, animal experiments, and statistical analysis. HU performed virus preparation and participated in the animal experiments. MO designed the study, analyzed the nucleotide sequences, and drafted the manuscript. All authors read and approved the final manuscript.
